# A Transgenic Rat for Investigating the Anatomy and Function of Corticotrophin Releasing Factor Circuits

**DOI:** 10.3389/fnins.2015.00487

**Published:** 2015-12-24

**Authors:** Matthew B. Pomrenze, E. Zayra Millan, F. Woodward Hopf, Ronald Keiflin, Rajani Maiya, Angelo Blasio, Jahan Dadgar, Viktor Kharazia, Giordano De Guglielmo, Elena Crawford, Patricia H. Janak, Olivier George, Kenner C. Rice, Robert O. Messing

**Affiliations:** ^1^Division of Pharmacology and Toxicology, College of Pharmacy, The University of Texas at AustinAustin, TX, USA; ^2^Department of Neurology, University of California, San FranciscoSan Francisco, CA, USA; ^3^Committee on The Neurobiology of Addictive Disorders, The Scripps Research InstituteLa Jolla, CA, USA; ^4^Chemical Biology Research Branch, Drug Design and Synthesis Section, National Institute on Drug Abuse, National Institute on Alcohol Abuse and AlcoholismRockville, MD, USA

**Keywords:** Cre recombinase, CRF, channelrhodopsin-2, designer receptors exclusively activated by designer drugs, transgenic rat models, central amygdala, Fos, R121919

## Abstract

Corticotrophin-releasing factor (CRF) is a 41 amino acid neuropeptide that coordinates adaptive responses to stress. CRF projections from neurons in the central nucleus of the amygdala (CeA) to the brainstem are of particular interest for their role in motivated behavior. To directly examine the anatomy and function of CRF neurons, we generated a BAC transgenic *Crh*-Cre rat in which bacterial Cre recombinase is expressed from the *Crh* promoter. Using Cre-dependent reporters, we found that Cre expressing neurons in these rats are immunoreactive for CRF and are clustered in the lateral CeA (CeL) and the oval nucleus of the BNST. We detected major projections from CeA CRF neurons to parabrachial nuclei and the locus coeruleus, dorsal and ventral BNST, and more minor projections to lateral portions of the substantia nigra, ventral tegmental area, and lateral hypothalamus. Optogenetic stimulation of CeA CRF neurons evoked GABA-ergic responses in 11% of non-CRF neurons in the medial CeA (CeM) and 44% of non-CRF neurons in the CeL. Chemogenetic stimulation of CeA CRF neurons induced Fos in a similar proportion of non-CRF CeM neurons but a smaller proportion of non-CRF CeL neurons. The CRF1 receptor antagonist R121919 reduced this Fos induction by two-thirds in these regions. These results indicate that CeL CRF neurons provide both local inhibitory GABA and excitatory CRF signals to other CeA neurons, and demonstrate the value of the *Crh*-Cre rat as a tool for studying circuit function and physiology of CRF neurons.

## Introduction

Corticotrophin-releasing factor (CRF) is a central regulator of endocrine, autonomic, and behavioral responses to stressors (Koob, 2009). Although CRF cell bodies are distributed in several brain regions, they are particularly concentrated in the central amygdala (CeA), the bed nucleus of the stria terminalis (BNST), and the paraventricular hypothalamic nucleus (PVN; Wang et al., 2011). In the PVN CRF acts as a hormone to regulate the hypothalamic-pituitary-adrenal (HPA) axis and trigger the endocrine stress response (Rivier and Vale, 1983). Outside of the PVN CRF modulates synaptic transmission within specific circuits of the central nervous system (Gallagher et al., 2008). CRF neurons of the CeA are of particular interest, since they contribute to stress-related arousal, conditioned fear, and negative emotional states associated with drug withdrawal (Koob, 2009; Walker et al., 2009; Kravets et al., 2015).

In the rat, the CeA subpopulation that expresses CRF resides in the lateral CeA (CeL) where another, mostly non-overlapping subpopulation expresses enkephalin (Veinante et al., 1997; Day et al., 1999). Approximately 60% of CeL CRF neurons are also immunoreactive for dynorphin (Marchant et al., 2007). Anatomical studies have shown strong projections from the CeL as a whole to the medial CeA (CeM), the brainstem (parabrachial nucleus, reticular formation, locus coeruleus, nucleus of the solitary tract and dorsal vagal complex) and the BNST, with more modest projections to the lateral hypothalamus, lateral one-third of the substantia nigra pars compacta and an adjacent lateral part of the retrorubral field (Petrovich and Swanson, 1997; Zahm et al., 1999; Bourgeais et al., 2001; Dong et al., 2001). For CeL CRF neurons in particular, tract-tracing studies have identified CRF projections from the rat CeA to the locus coeruleus (Van Bockstaele et al., 1998; Reyes et al., 2011), parabrachial nuclei (Moga and Gray, 1985), the midbrain central gray (Gray and Magnuson, 1992), the dorsal vagal complex [including the nucleus tractus solitarius (NTS)] (Gray and Magnuson, 1987), the pontine reticular nucleus (Fendt et al., 1997), the mesencephalic trigeminal nucleus (Sakanaka et al., 1986), and the BNST (Sakanaka et al., 1986). Whether CeL CRF neurons also project locally within the CeA is not clear, and although some CRF immunoreactive fibers have been observed in the CeM (Veening et al., 1984), their source and functional significance are not known. Several recent studies have helped clarify CRF architectures and functions using *Crh*-Cre mouse lines (Gafford et al., 2012, 2014; Wamsteeker Cusulin et al., 2013; McCall et al., 2015), but thorough characterization of CRF circuits across brain structures, and moreover across species, is still lacking.

Here, we describe a transgenic *Crh*-Cre rat that permits genetic access to CRF neurons, thereby allowing direct investigation of their anatomy and roles in physiology and behavior. To examine CRF cell localization and projection targets, we crossed *Crh*-Cre rats with a DsRed2/GFP-reporter rat, or infected the CeA with AAVs that express Cre-dependent mCherry, channelrhodopsin (ChR2)-eYFP, or hM3Dq-mCherry. We found that Cre-expressing CeA neurons are immunoreactive for CRF and project to several brain regions in the brainstem and diencephalon. Using the *Crh*-Cre rat to investigate CeL circuitry, we provide new evidence that CRF-expressing CeL neurons act as local interneurons to provide both inhibitory and excitatory signals to the CeL and CeM.

## Materials and methods

### Development of *Crh*-Cre rats

All animal studies were approved by the Institutional Animal Use and Care Committees of the Ernest Gallo Clinic and Research Center at the University of California San Francisco, the Scripps Research Institute and of The University of Texas at Austin, and were performed in adherence with the NIH Guide for Care and Use of Laboratory Animals. Studies utilized male and female *Crh*-Cre rats.

We identified the BAC clone CH230-206D8 from the CHORI-230 Rat (BN/SsNHsd/MCW) BAC library, which was derived from an inbred female brown Norway rat (Osoegawa et al., 2004), as containing the promoter region and exons 1 and 2 of the rat *Crh* gene on chromosome 2. The BAC clone has ~80 kb 3′ of the *Crh* ATG and ~143 kb of DNA 5′ of the ATG. BAC recombineering was performed as described (Cotta-de-Almeida et al., 2003) with vectors and bacterial host cell lines kindly provided by Dr. Scott Snapper (Harvard Medical School).

A ~2.7 Kb modified/enhanced Cre metallothionein-1 polyadenaylation (CREM) fragment was PCR amplified from the plasmid p210 pCMV-CREM (Addgene # 8395; Kaczmarczyk and Green, 2001). This fragment contains a modified human beta-globin intron within the Cre coding sequence to prevent expression of Cre recombinase in prokaryotes, thereby making it suitable for interim work in bacteria with plasmids containing loxP sites. The fragment was sub-cloned into the conditional replicon vector pBSB-171 and confirmed by sequencing. The plasmid pBSB-171 allows the cloning of the fragments of interest and contains a floxed aminoglycoside kinase (aph) gene cassette. A c-myc tag (EQKLISEEDL) was inserted (QuikChange II XL Site Directed Mutagenesis kit, Agilent Technologies) immediately before the Cre “stop” codon, and confirmed by sequencing. The completed construct is referred to as CREM-myc pBSB-171.

We designed a forward PCR primer (P1) containing 58 bp homologous to the rat *Crh* sequence immediately adjacent to the ATG of CRF followed by 31 bp of CREM-myc pBSB-171 vector sequence, and a backward PCR primer (P2) containing 23 bp of CREM-pBSB-171 vector sequence followed by 64 bp homologous to the sequence immediately adjacent to the *Crh* stop codon. We then amplified a fragment containing the CREM-myc-floxed aph cassette with rat Crh homology ends by PCR with CREM-myc pBSB-171 and primers P1, P2. Lambda red-driven recombination between this PCR product and the BAC clone CH230-206D8 generated recombinants in which the endogenous Crh coding sequence was replaced with the CREM-myc-floxed aph fragment.

This recombineered CH230-206D8 BAC was transformed with the bacterial Cre expression plasmid 706-Cre;tet (Gene Bridges Gmbh, Heidelberg, Germany) to remove the floxed aph cassette. The recombineered circular BAC DNA without the aph cassette was purified (Bimboim and Doly, 1979) using a NucleoBond BAC 100 kit (Clontech # 740579). This DNA was sent to The University of Michigan Transgenic Animal Model Core for pronuclear injection into Hdr:W1 ES cells and implantation (Filipiak and Saunders, 2006). Rat-tail DNA from resulting progeny was purified using DNeasy (Qiagen # 69506), screened by PCR, and confirmed by sequencing to identify a total of three founder transgenic rats. Cre-expressing cells were identified by crossing *Crh*-Cre rats with the reporter rat line W-Tg(CAG-DsRed2/GFP) 15Jms (NBRP-Rat Number 0282), which was obtained from the National BioResource Project-Rat in Kyoto, Japan. The reporter rat has a DsRed coding region flanked by LoxP sites followed by a GFP sequence, all under control of a CAG promoter. Cre recombination leads to excision of the DsRed coding region and expression of GFP.

### Surgery and histology

We microinjected 0.8–1.2 μL/side (100 nL/min) of one of the following: AAV-Ef1α-DIO-eYFP, AAV-Ef1α-DIO-ChR2-eYFP (Zhang et al., 2010), AAV-hSyn-DIO-mCherry (UNC Vector Core, Chapel Hill, NC), AAV-hSyn-DIO-hM3Dq-mCherry, or AAV-hSyn-DIO-hM4Di-mCherry (Krashes et al., 2011). Coordinates for the CeA were AP 2.40, ML ± 4.85, DV −8.40 from the skull in adult rats, or AP −2.0, ML ±4.3, DV −7.9 from the skull in adolescent rats weighing 200–220 g. Coordinates for the BNST were AP +0.00, ML ±3.5, DV −6.8 with a 16° angle in adolescent rats weighing 200–220 g. After injection, we waited 10 min for virus to diffuse into the tissue before retracting the injector needle. We used adolescent rats in several experiments to facilitate transduction down axons for efficient labeling of neuronal projections. After 2–4 months, rats were deeply anesthetized with sodium pentobarbital (100 mg/kg, ip) and perfused transcardially with phosphate buffered saline (PBS) followed by 4% paraformaldehyde in PBS. Brains were immediately removed, placed into the same fixative overnight, and then transferred to a 30% sucrose solution at 4°C before sectioning at 40 μm on a cryostat.

We detected co-localization of eYFP or mCherry fluorescence with CRF, prodynorphin, preproenkephalin, somatostatin, protein kinase C delta (PKCδ), or Fos immunoreactivity using immunofluorescent histochemistry. Sections were washed three times in PBS with 0.2% Triton X-100 (PBST) for 10 min at room temperature and then incubated in blocking solution made of PBST with 3% normal donkey serum (Jackson ImmunoResearch, number 017-000-121) or normal goat serum (Jackson ImmunoResearch, number 005-000-121) for 1 h. Sections were then incubated in one or more of the following primary antibodies: rabbit anti-cFos (1:2000, Santa Cruz Biotechnology, sc-52), goat anti-cFos (1:2000, Santa Cruz Biotechnology, sc-52-G), mouse anti-tyrosine hydroxylase (TH; 1:2000, Immunostar, 22941), mouse anti-tryptophan hydroxylase (TPH; 1:1000, Sigma Aldrich, T0678), goat anti-CRF (1:500-1000, Santa Cruz Biotechnology, sc-1761 Lot# B0315), guinea pig anti-prodynorphin (1:500, Neuromics, GP10110), rabbit anti-preproenkephalin (1:100, Neuromics, RA14124), or rabbit anti-PKCδ (1:2000, Santa Cruz Biotechnology, sc-213) with or without mouse anti-NeuN (1:2000, Millipore, MAB377 clone A60) in blocking solution rotating at 4°C for 18–20 h. After three 10-min washes in PBST, sections were incubated in species-specific secondary antibodies Alexa Fluor 488, 568, or 647 (1:700, Thermo-Fisher Scientific, A-21206, A11067, A-11055, A-21202, A- 21208, A-11073, A-21447, A-31573) in blocking solution for 1 h at room temperature. Finally, sections were washed four times in PBS, then mounted in 0.2% gelatin water onto SuperFrost Plus glass slides (Fisherbrand, 12-550-15) and coverslipped with Fluoromount-G (Southern Biotech, 0100-01). Slides were stored in the dark before microscopy and image acquisition.

For somatostatin staining, sections were pretreated with 50% ethanol twice for 10 min each and washed three times in PBS and then blocked in 10% normal donkey serum at room temperature for 10 min. The sections were then incubated with rat anti-somatostatin antibody (Millipore, MAB354) diluted 1:100 in PBS containing 0.05% Triton-X-100 and incubated for 20 h at 4°C with shaking. Sections were washed for 10 min three times in PBS and then incubated with 2% NDS for 10 min. Primary antibody staining was visualized by incubating with Alexa Fluor 488-conjugated anti-rat secondary antibody (1:700 dilution in PBS) for 2 h. Sections were washed four times in PBS and prepared for imaging as described above.

Peroxidase immunohistochemistry was performed using 3,3′-diaminobenzidine tetrahydrochloride (DAB). Sections were first washed for 30 min each in 0.1 M sodium phosphate buffer, pH 7.2 (PB), followed sequentially by 50% ethanol, 50% ethanol with 3% H_2_O_2_, and then 5% normal donkey serum (NDS) in PB. Sections were then incubated in mouse antiserum against GFP (1:1500, Invitrogen or 1:1000, Abcam) in PB with 2% NDS and 0.2% Triton X-10 (PBTX; 48 h at 4°C). The sections were then washed with PB and incubated in PBTX containing biotinylated donkey anti-mouse IgG (1:1000, Jackson Immunoresearch Laboratories) for 24 h at 4°C. Finally, sections were washed in PB, incubated with peroxidase-conjugated avidin (ExtrAvidin, Sigma-Aldrich) in PB (1:2500; 2 h at RT), washed again, and then incubated in DAB (ImmPACT DAB, Vector Laboratories). Sections were then mounted with PB containing 1% gelatin, dehydrated, cleared in xylene and coverslipped with DEPEX mounting medium (Electron Microscopy Sciences).

### Confocal acquisition and 3D analysis

Three-dimensional stacks of Images were acquired with a 780 Laser Scanning Confocal microscope (Zeiss, Inc.) using either a 20x (1 μm image slice), 40x (0.6 μm image slice), or 63x (0.2 μm image slice) objective. The system is equipped with a stitching stage and Zen software to reintegrate the tiled image stacks. Stitched z series images of the entire CeA were imported into Imaris software (Bitplane-Andor, Inc.) for quantitation. The eYFP (green), and the CRF-A568 fluorescent labels (red) were first three dimensionally traced using the iso-surfacing module to obtain a clean outline of the neuronal cell body and branches, which was then rendered solid using a control based threshold. The isosurfaced eYFP signal was then used to create a new channel to determine how much red signal was present within green iso-surfaced regions. This assay was further corroborated using the colocalization module to confirm the extent and location of overlapping signals. The filament tracer module was used to identify the origin of disjointed axons and outline the neural branches of the same neuron. An alternative analysis of green fluorescent signals within red iso-surfaced neurons was performed for comparison. This use of multiple methods of analysis allowed us to quantify the location and extent of CRF-like immunoreactivity throughout eYFP positive cell bodies and axons. This approach was used on 1–3 sections per rat from five rats.

### Cell counting

Immunostained sections were imaged on a Zeiss 710 LSM confocal microscope, Zeiss Imager M2 microscope, or a Zeiss Axio Zoom stereomicroscope. Quantification of Fos and co-localization of -mCherry or eYFP with neuropeptides in the CeA were performed on alternate sections from Bregma −1.90 to −3.00 (6–12 sections per rat) using Fiji (Schindelin et al., 2012).

### Electrophysiology and optogenetics

To measure ChR2-evoked GABA IPSCs, we expressed ChR2-eYFP in Cre-expressing neurons and recorded light-evoked IPSCs as in recent work (Seif et al., 2013), with the following exceptions: rats were perfused intracardially with a glycerol-based aCSF (in mM: 252 glycerol, 2.5 KCl, 1.25 NaH_2_PO_4_, 1 MgCl_2_, 2 CaCl_2_, 25 NaHCO_3_, 1 L-ascorbate, and 11 glucose), and then brain slices were cut in the same solution. A CsCl internal solution (CsCl, 135; HEPES, 10; MgATP, 4; GTP, 0.3; MgCl_2_, 2; EGTA, 0.5 and QX-314, 5; pH 7.23, Naylor et al., 2010), with DNQX in the bath to block AMPARs, was used for measuring GABA IPSCs.

To distinguish small, evoked IPSCs from spontaneous IPSCs (sIPSCs), we recorded ~20 traces where a ChR2-eYFP+ terminal was stimulated once with blue light at 111 ms into a 1000 msec sweep. The sIPSC frequency was typically low (0.67 ± 0.13 Hz), and thus the likelihood of observing a spontaneous IPSC exactly at the time of ChR2 stimulation in more than a few of the 20 traces was very low. If an IPSC was observed at the time of ChR2 stimulation in only one or two of the 20 traces in a given cell, we did not consider this a cell responding to ChR2 stimulation. Of note, sIPSCs displayed variability in amplitude within the same cell as reported by others (Delaney and Sah, 2001), making the relative amplitude of evoked vs. spontaneous IPSCs a less reliable measure. For spatial mapping, we used live visualization of the electrode tip and its exact location within the CeA instead of biocytin filling. Had we used biocytin in these experiments, there would have been many neurons filled within the same slice, including neurons where patch-clamping was attempted for several minutes but failed to achieve stable recording.

### Chemogenetics and Fos mapping

*Crh*-Cre rats were microinjected bilaterally with AAV-hSyn-DIO-hM3Dq-mCherry, AAV-hSyn-DIO-hM4Di-mCherry, or AAV-hSyn-DIO-mCherry into the CeL. After 2–4 months, rats were administered intraperitoneally 2 mg/kg clozapine-N-oxide (CNO; NIMH Chemical Synthesis and Drug Supply Program) and perfused 120 min later for Fos immunohistochemistry. To inhibit CRF1 receptors, we administered 10 mg/kg R121919 (Chen et al., 2004) subcutaneously to rats 30 min before administration of CNO.

### Data analysis

Data are shown as mean ± SEM values and were analyzed by two-tailed *t*-tests or by ANOVA with *post-hoc* Tukey's tests using GraphPad Prism v6.0.

## Results

### Neurons that express Cre in *Crh*-Cre rats are immunoreactive for CRF

**Figure 1:**
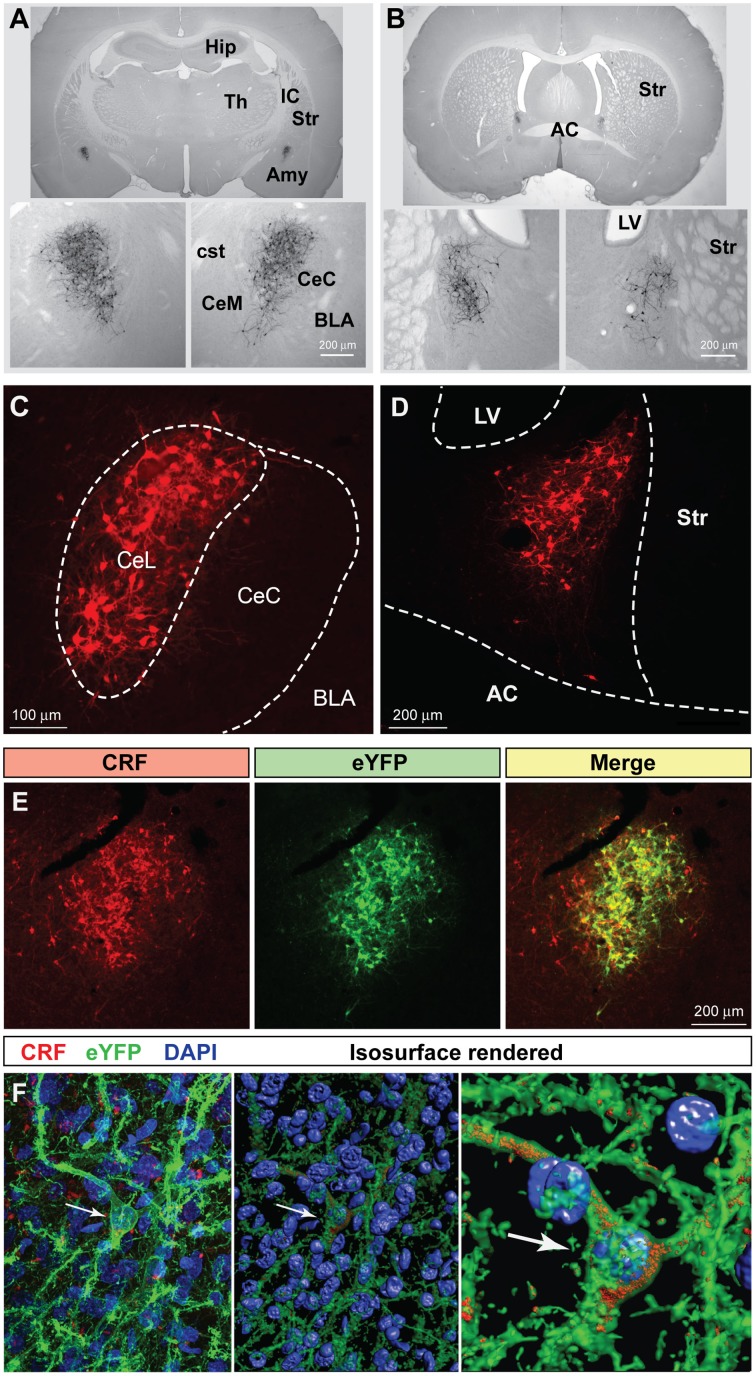
***Crh*-Cre rats express Cre recombinase activity in the CeL and dlBNST**. **(A,B)** Bigenic progeny of a *Crh*-Cre X DsRed2/GFP cross display robust GFP labeling in the CeL and dlBNST. Scale bars, 200 μm. **(C,D)** Cre-dependent mCherry expression in Cre-expressing neurons of the CeL and dlBNST. Scale bars, 100 μm in panel **(C)**; 200 μm in panel **(D)**. **(E)** Cre-dependent eYFP co-localizes with CRF immunoreactivity in the CeL. Scale bar, 200 μm. **(F)** Rendered isosurface analysis demonstrates co-localization of CRF immunoreactivity within CeL neurons that also express Cre-dependent eYFP. Arrows point to an example of eYFP and CRF in the same neuron. AC, anterior commissure; AMY, amygdala; BLA, basolateral amygdala; CeC, capsular central amygdala; CeM, medial central amygdala; cst, commissural stria terminalis; Hip, Hippocampus; IC, internal capsule; LV, lateral ventricle; Str, Striatum; Th, Thalamus.

Cre-expressing cells in *Crh*-Cre rats were first identified by crossing *Crh*-Cre rats with W-Tg(AG-DsRed2/GFP)15Jms reporter rats and then immunostaining brain sections from bigenic progeny with anti-GFP antibody. There were clusters of immunoreactive neurons in the CeL and in the dorsolateral BNST (Figures 1A,B). We confirmed the presence of Cre activity in mature CeL and BNST neurons by microinjecting AAV-hSyn-DIO-mCherry into the central amygdala or the dorsal BNST of 6 week-old rats and then examining brain slices for the presence of mCherry fluorescence 8 weeks later (Figures 1C,D). Surprisingly we did not detect Cre recombination in the ventral BNST or in the paraventricular hypothalamic nucleus, even following microinjection of a large volume (1.2 μl) of Cre-dependent AAV into the hypothalamus.

To determine if Cre-expressing neurons also express CRF, we microinjected colchicine (4 μg in 0.8 μL) into the CeA 4 weeks after injection of AAV-Ef1α-DIO-eYFP and 72 h prior to perfusion to allow CRF to accumulate in the cell soma (Merchenthaler, 1984). We found that 94.6 ± 1.2% eYFP+ neurons were immunoreactive for CRF, while 77.1 ± 2.1% of CRF immunoreactive neurons expressed eYFP (Figure 1E; *n* = 3 rats, 12 amygdala sections/rat).

We also examined colocalization of ChR2-eYFP and CRF immunoreactivity in *Crh*-Cre rats microinjected with AAV-Ef1α-DIO-ChR2-eYFP and not treated with colchicine. ChR2-eYFP was present in the cell membrane of neuronal cell bodies and processes while CRF immunoreactivity was mainly scattered within neural processes. Because CRF is mainly localized in neural processes, quantification of colocalization in cell bodies using bright-field microscopy at 40x resulted in only a small percentage (16.5 ± 2.7%) of eYFP+ neurons being colocalized with CRF. However, confocal analysis at 63x followed by 3D reconstruction of the neuronal cell bodies and branches using Imaris 3D software revealed that all eYFP+ neurons contained CRF immunoreactivity in the cell soma or branches (Figure 1F). Out of 155 neurons analyzed, 100 ± 0% of eYFP + neurons were positive for CRF, while 99.4 ± 0.6% of CRF+ neurons were positive for eYFP.

**Figure 2:**
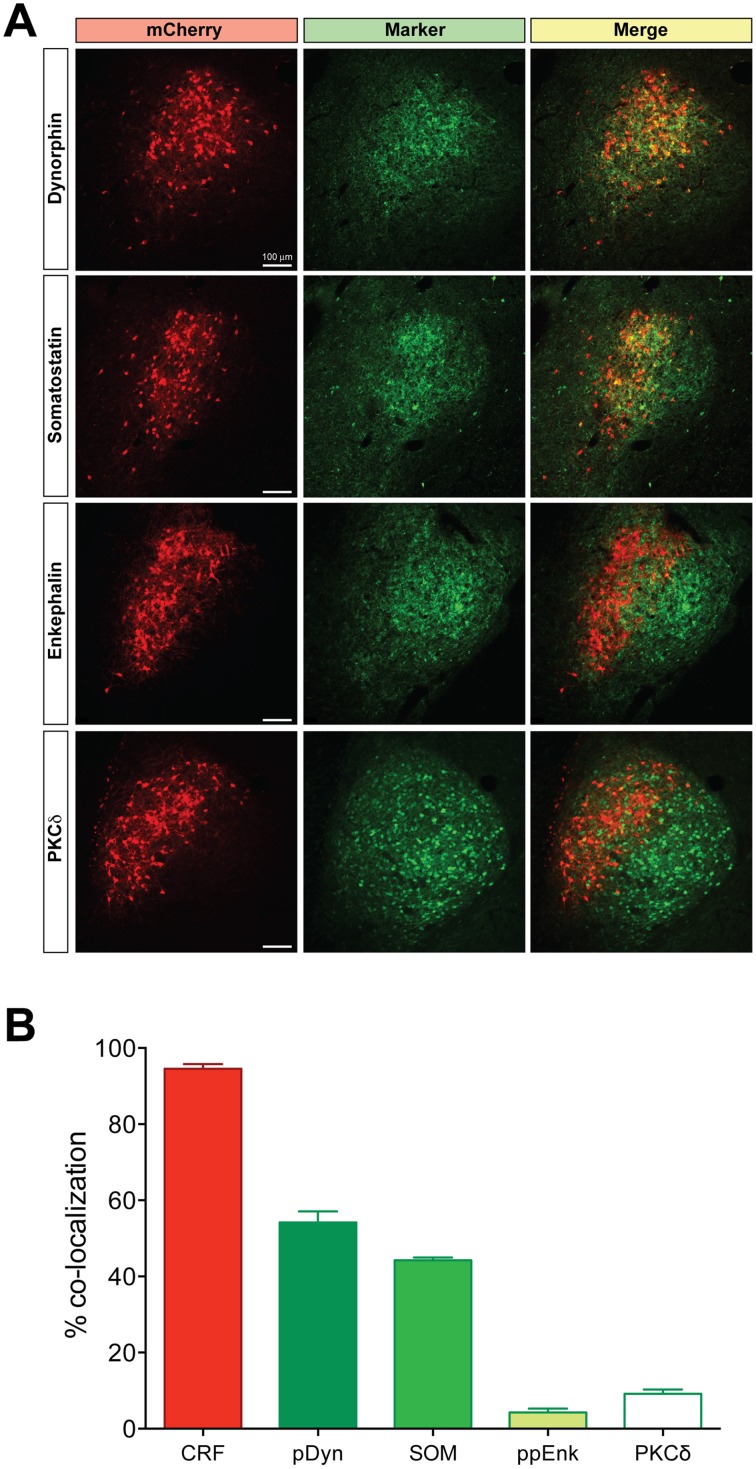
**Coexpression of other neuropeptides in CeL CRF neurons**. **(A)** A large percentage of CRF neurons identified by expression of Cre-dependent mCherry are immunoreactive for dynorphin and somatostatin, while few express enkephalin or PKCδ. Scale bars, 100 μm. Medial is to the left. **(B)** Quantification of mCherry expression with neuropeptide immunoreactivity; CRF *n* = 3 rats, 12 amygdala sections/rat; dynorphin *n* = 4 rats, 6 amygdala sections/rat; somatostatin *n* = 4 rats, 6 amygdala sections/rat; enkephalin *n* = 4 rats, 6 amygdala sections/rat; PKCδ, *n* = 6 rats, 10–12 amygdala sections/rat.

Using hSyn-DIO-mCherry to identify CRF neurons, we examined co-expression of other neuropeptides in the CeL (Figure 2). We found that about 54.2 ± 2.8% of CRF neurons were immunoreactive for dynorphin while there was almost no colocalization with enkephalin, as described previously (Veinante et al., 1997; Day et al., 1999; Marchant et al., 2007). A population of neurons in the CeL expresses somatostatin (SOM), and recent studies demonstrate an active role for CeL SOM+ neurons in conditioned fear in mice (Li et al., 2013; Penzo et al., 2015). We determined that approximately 44.2 ± 0.7% of CeL CRF neurons co-localize with SOM+ neurons. In addition to SOM+ neurons, there is a distinct GABA-ergic subpopulation of CeL neurons in mice that expresses protein kinase C delta (PKCδ), but not CRF, and suppresses fear conditioning (Ciocchi et al., 2010; Haubensak et al., 2010). We similarly found that CRF and PKCδ are present in distinct populations in the rat amygdala with only approximately 9.2 ± 1.1% of CRF neurons co-expressing PKCδ. Also, CRF-expressing cells were consistently more medial than PKCδ-expressing cells in the CeL (Figure 2A).

### Projections from CeL CRF neurons outside the CeA

**Figure 3:**
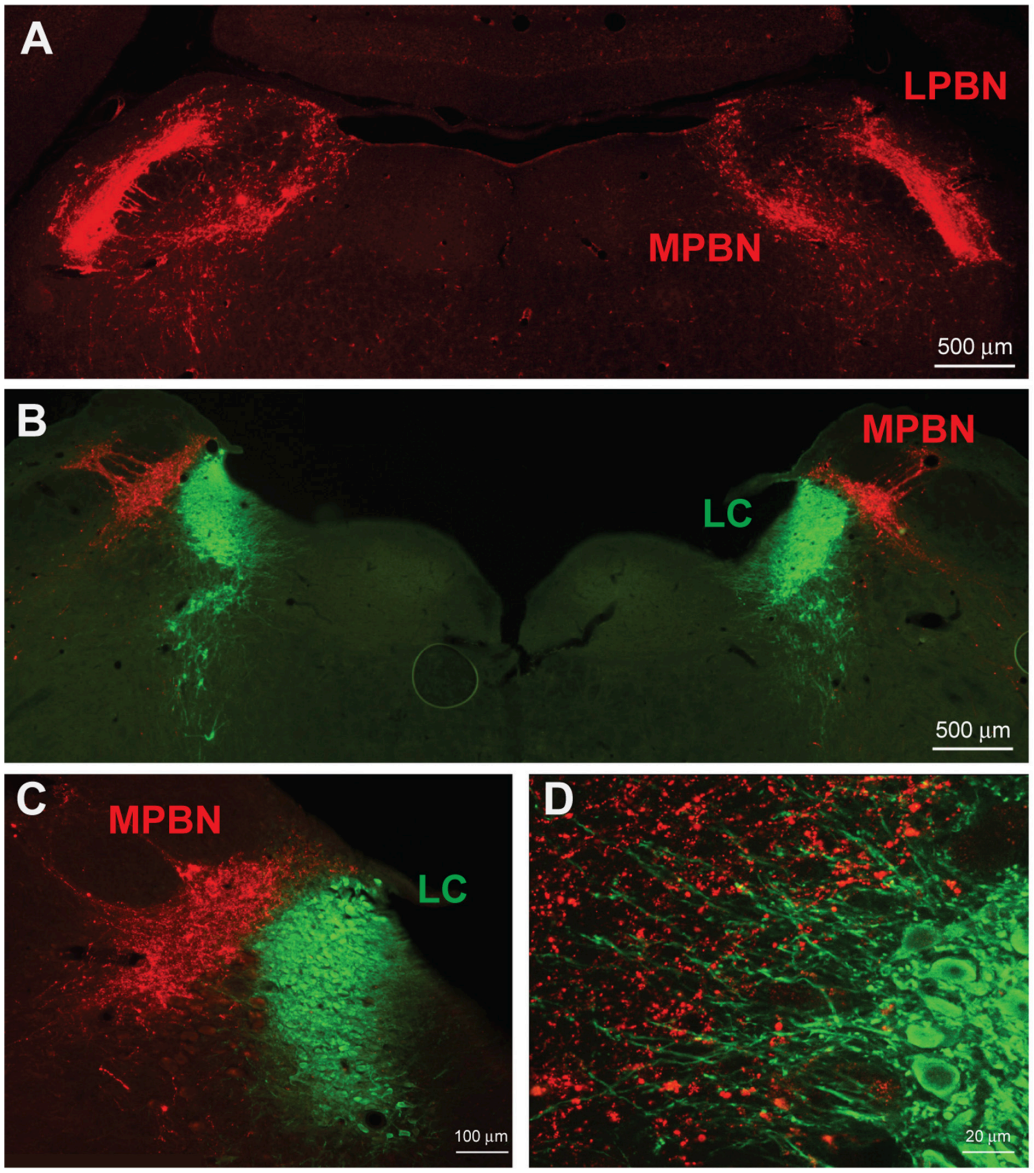
**CeL CRF neurons project strongly to brainstem nuclei**. **(A)** After injection of AAV-hSyn-DIO-mCherry into the CeL, mCherry expressing fibers were detected in the lateral and medial parabrachial nuclei (Bregma −9.0). Scale bar, 500 μm. **(B)** mCherry expressing fibers were also detected in the medial parabrachial nucleus just lateral to the locus coeruleus (Bregma -9.6). Red, mCherry; Green, Tyrosine Hydroxylase. Scale bar, 500 μm. **(C,D)** High-magnification examples of mCherry fibers from the CeL and noradrenergic LC neurons. CeL fibers appear to run orthogonally to noradrenergic dendrites extending laterally from the LC core into the medial parabrachial nucleus. Scale bars, 100 μm in panel **(C)**; 20 μm in panel **(D)**. MPBN, medial parabrachial nucleus; LPBN, lateral parabrachial nucleus; LC, locus coeruleus.

**Figure 4:**
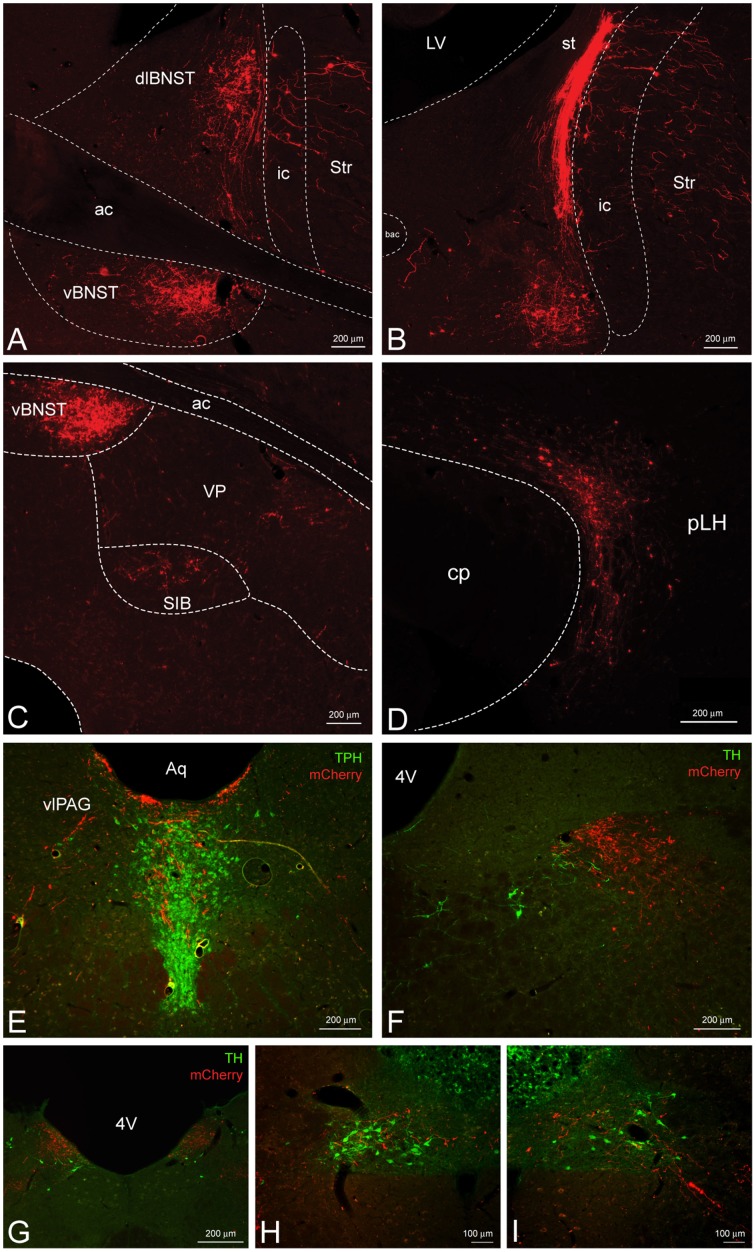
**CeL CRF neurons provide inputs to other limbic brain structures**. **(A,B)** A dense bundle of mCherry expressing fibers from the CeL were observed in the dorsolateral and ventral BNST. **(A)** Rostrally, fibers clustered mainly in the oval nucleus of the dorsal bed nucleus and in the subcommisural zone of the ventral bed nucleus (Bregma −0.12). **(B)** Caudally, dense fibers of the stria terminalis were present in the dorsal region (Bregma −0.6). Scale bars, 200 μm. **(C)** Less dense projections were detected ventral and lateral to the ventral BNST in the substantia innominata and the ventral pallidum (Bregma −0.12). Scale bar, 200 μm. **(D)** Fibers were detected throughout the lateral hypothalamus (Bregma -4.20) within the nigrostriatal bundle. Scale bar, 200 μm. **(E)** Fibers also projected dorsomedially into the caudal dorsal raphe nucleus and ventrolateral periaqueductal gray (Bregma −7.7). TPH, tryptophan hydroxylase. Scale bar, 200 μm. **(F–I)** Some fibers projected as far as the nucleus tractus solitarius where they came in close contact to noradrenergic processes and cell bodies in the most caudal regions. Bregma (−12.9) – (−14.0). TH, tyrosine hydroxylase. Scale bars, 200 μm in panel **(F)**; 200 μm in panel **(G)**; 100 μm in panel **(H)**; 100 μm in panel **(I)**. ac, anterior commissure; ic, internal capsule; Str, striatum; st, stria terminalis; VP, ventral pallidum; SIB, substantia innominata; cp, cerebral peduncle; pLH, posterior lateral hypothalamus; Aq, central aqueduct; vlPAG, ventrolateral periaqueductal gray; 4V, fourth ventricle.

**Figure 5:**
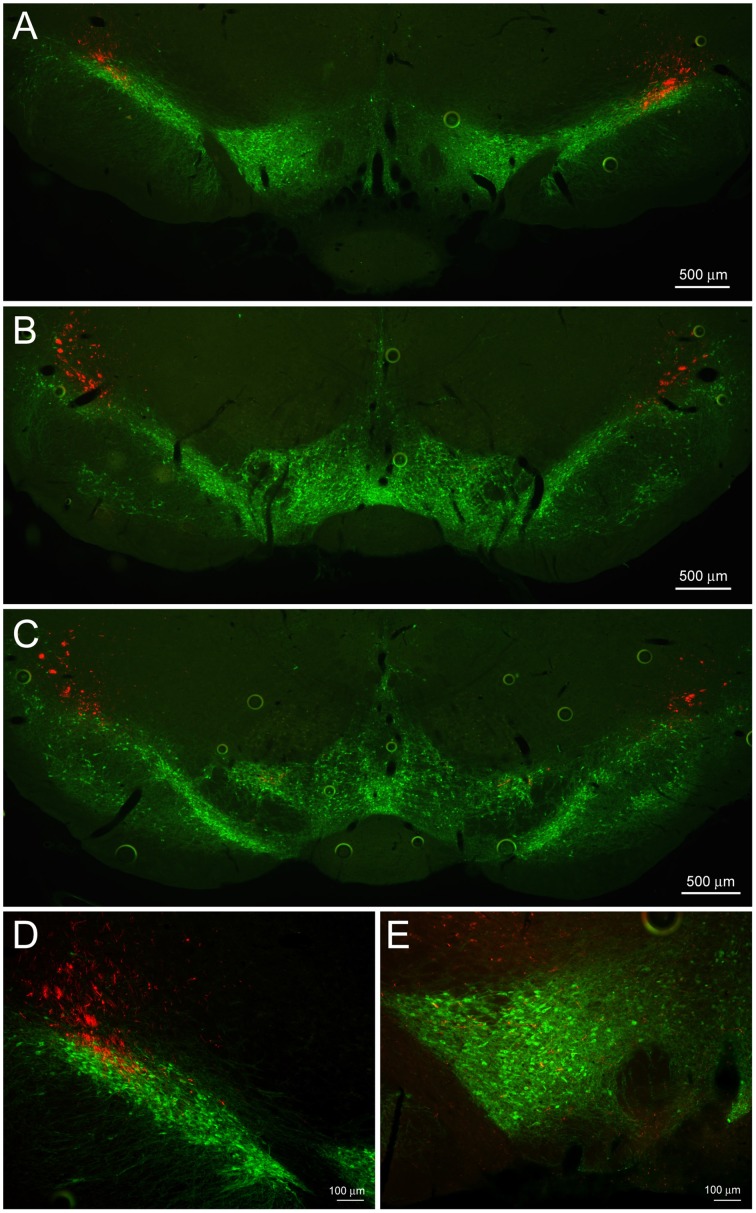
**CeL CRF projections to the substantia nigra and VTA**. **(A)** Representative example of CeL CRF fibers in the rostral VTA and substantia nigra pars compacta (SNc; Bregma −5.0). Scale bar, 500 μm. **(B)** CeL CRF fibers were observed projecting through the SNc, but not contacting the VTA slightly more caudally (Bregma -5.5). Scale bar, 500 μm. **(C)** CeL CRF fibers were present at the most caudal aspects of the VTA and SNc (Bregma −6.1). Scale bar, 500 μm. **(D)** CeL CRF fibers course through the most dorsolateral region of the SNc. Scale bar, 100 μm. **(E)** Low density collaterals were present in the rostral VTA surrounding dopamine neurons. Scale bar, 100 μm. Green, tyrosine hydroxylase.

**Figure 6:**
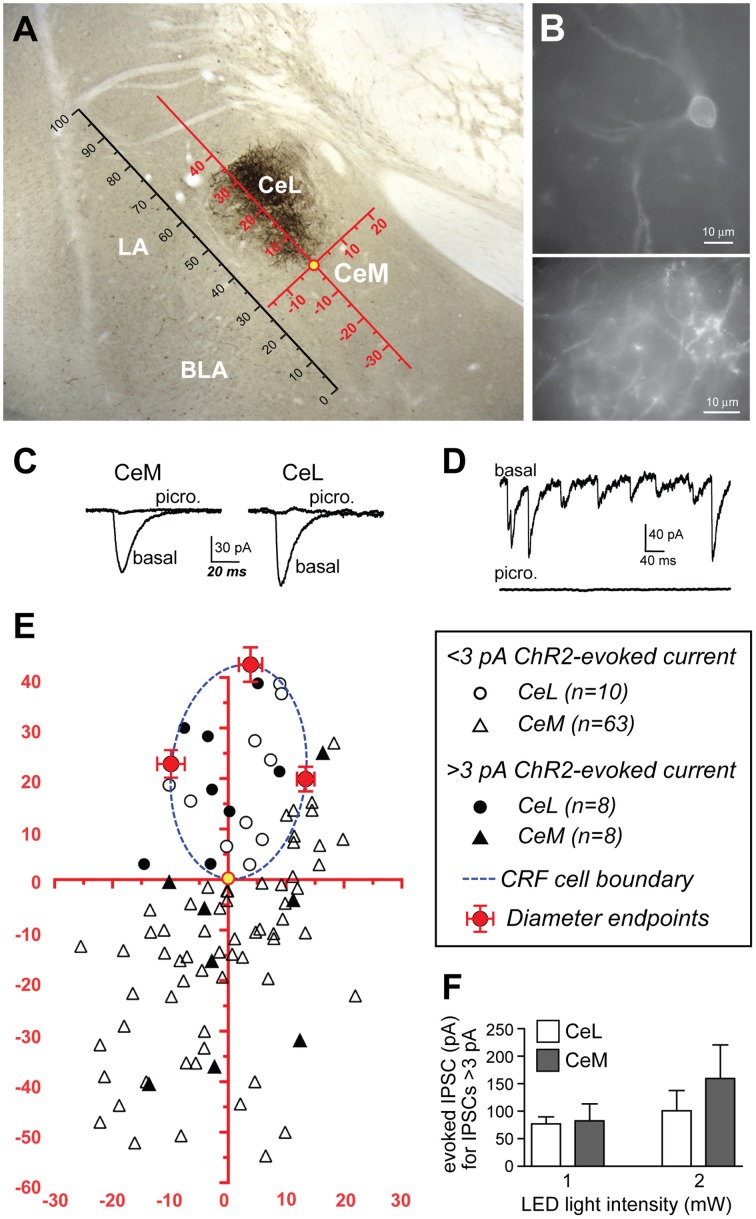
**ChR2 stimulation of CRF CeL terminals evokes IPSCs in a subset of CeA neurons**. **(A)** Coronal section through the CeA with superimposed reference frame to identify the location of recorded neurons across slices. **(B)** Example of live CeL neurons (Scale bar, 10 μm) and fibers (Scale bar, 50 μM) expressing ChR2-eYFP. **(C)** Examples of IPSCs evoked after stimulation of ChR2 in CRF CeL inputs, which were blocked by picrotoxin. **(D)** Picrotoxin also blocked spontaneous IPSCs. **(E)** Diagram showing distribution of recorded cells relative to the cluster of CRF cell bodies and dendrites in the CeL (dotted blue circle). Filled symbols represent neurons with IPSC responses; open symbols are neurons without evoked IPSC responses. One CeL neuron with an IPSC response was found outside the cluster of CRF cell bodies and dendrites but within the confines of the CeL. **(F)** Similar magnitude of evoked IPSCs in CeL and CeM neurons and at two different LED intensities. BLA, basolateral amygdala; CeL, lateral central amygdala; CeM, medial central amygdala; LA, lateral amygdala.

We examined neuronal projections from CeL CRF neurons using mCherry or ChR2-eYFP as a histological marker (Figures 3–**6**). We detected projections to several regions identified previously in nonselective tract tracing studies of the CeL (Petrovich and Swanson, 1997; Veinante and Freund-Mercier, 1998; Zahm et al., 1999; Bourgeais et al., 2001; Dong et al., 2001). The largest and densest were to the PBN and the LC (Figure 3). Fibers were present in both the lateral and medial PBN and in the mesencephalic trigeminal nucleus, and extended caudally within the medial PBN to the LC. CRF fibers there appeared to be interdigitated and orthogonal to the dorsolateral LC dendritic field (Figure 3D).

We also observed a substantial projection from the CeL to the dorsolateral and especially the ventral BNST (Figures 4A–C). Dorsolateral CRF fibers appeared to cluster around the oval nucleus and also extend into the adjacent dorsal striatum. In addition, a small projection was detected slightly ventrolateral to the ventral BNST in the substantia innominata and ventral pallidum (Figure 4C). Caudal to the BNST, CRF projections were present in the most lateral portion of the lateral hypothalamus (LH) along its entire anterior-posterior axis traveling through the nigrostriatal bundle (Figure 4D). Most of these appeared to be fibers of passage with small projections extending medially into the LH. Caudal to the hypothalamus, we observed CRF fibers coursing into the ventrolateral periaqueductal gray, and eventually into the caudal aspect of the serotonergic dorsal raphe nucleus (Figure 4E). Deep in the brainstem caudal to the LC, we detected a small projection to the nucleus tractus solitarius (NTS) throughout much of its anterior-posterior axis (Figures 4F–I). At the most anterior aspect, CRF fibers were localized to the lateral NTS and overlapped with tyrosine hydroxylase positive processes but not somata (Figure 4F). Further posterior, CRF fibers clustered within the medial NTS as it coursed toward the 4th ventricle (Figure 4G), and fibers eventually terminated in the caudal ventrolateral NTS around noradrenergic cell bodies (Figures 4H,I).

CRF signaling in the dopaminergic ventral tegmental area (VTA) has garnered much attention due to its significant role in relapse to drug seeking (Shalev et al., 2010). However, the source of CRF in the VTA has remained controversial (Grieder et al., 2014; Zhao-Shea et al., 2015). CRF fibers from the CeL were present traveling through the dorsolateral substantia nigra pars compacta, most likely as fibers of passage on their way to the brainstem (Figures 5A–D). However, upon closer examination we detected minor collateral projections within the rostral VTA (Figure 5E).

### Projections from CeL CRF neurons within the CeA

Since the CeL sends dense projections to the CeM (Petrovich and Swanson, 1997), we investigated whether CRF expressing CeL neurons contribute to these projections. Surprisingly, following microinjection of AAV-hSyn-DIO-mCherry into the CeA, we detected very few mCherry-expressing projections from the CeL to the adjacent CeM (Figures 1C,6A). We next used AAV-Ef1α-DIO-ChR2-eYFP to express ChR2-eYFP in CeL CRF neurons (Figure 6B) and to detect light-evoked inhibitory postsynaptic currents (IPSCs) in CeA neurons that did not express ChR2-eYFP. To compare data across animals and brain slices, we mapped the spatial position of each recorded neuron to a common reference frame. First, we established a scale using the intermediate capsule between the BLA and CeA as a guide. We defined the distance between where the intermediate capsule meets the external capsule and the ventral border of the BLA as equal to 100 relative units (Figure 6A). We then mapped the position of each cell onto a common Cartesian coordinate system with the Y axis parallel to the intermediate capsule and the origin at the most ventral part of the ovoid cluster of CeL CRF cell bodies (Figures 6A,E). The position of each neuron was expressed as relative units along both axes. To assess the accuracy of this method, we mapped the position and size of the CRF cell body cluster in slices from 12 rats. We found that the coordinates of points defined by the intersection of the oval border of the CeL CRF cell body cluster with its maximal dorsal-ventral and medial-lateral diameters were consistent across slices (Figure 6E). The borders of the CeL CRF cell body cluster were also consistent when expressed in relative units using the bottom of the intermediate capsule as the origin for the coordinate system (bottom X: 22.9 ± 2.2, Y: 19.7 ± 1.8; top X: 27.0 ± 2.0, Y: 55.0 ± 1.8; medial edge X: 35.5 ± 2.1, Y: 44.0 ± 1.5; lateral edge X: 13.7 ± 1.4, Y: 41.3 ± 1.3).

Stimulation of ChR2 evoked IPSCs in 8 of 18 (44.4%) non-CRF CeL neurons. In contrast only 8 of 71 (11.3%) of CeM neurons demonstrated an IPSC, concurring with our histological findings of sparse mCherry fluorescence and eYFP immunoreactivity of fibers within the CeM (Figures 1C,6A). Picrotoxin (100 μM) blocked light-evoked IPSCs more than 97.7 ± 0.9% (*n* = 4; Figure 6C), as well as blocking spontaneous IPSCs (Figure 6D), which is consistent with previous studies demonstrating that CeA CRF neurons are GABA-ergic (Veinante and Freund-Mercier, 1998; Cassell et al., 1999; Day et al., 1999). The CeM neurons exhibiting light-evoked IPSCs were scattered rather than clustered in a subregion of the CeM (Figure 6E). The IPSC amplitudes were not different between CeM and CeL neurons at 2 vs. 1 mW of LED illumination (Figure 6F), indicative of a weak input-output relationship for ChR2 as described (Stuber et al., 2011). Together, these results suggest that CRF inputs target a small subset of CeM neurons. Nearly all CeM neurons showed spontaneous ISPCs that were greatly inhibited by picrotoxin (Figure 6D), demonstrating that most CeM neurons can respond to synaptically released GABA. Many cells also exhibited electrically evoked IPSCs, with kinetics similar to those seen for spontaneous IPSCs and ChR2-evoked IPSCs (Table 1) and previously reported for CeA IPSCs (Delaney and Sah, 2001; Naylor et al., 2010). Thus, our optogenetic results indicate that CeL CRF neurons send GABA-ergic projections to almost half of their neighboring non-CRF neurons in the CeL, but only to a small number of neurons in the CeM.

**Table 1:** **IPSC kinetics for spontaneous and evoked IPSCs in CeM neurons**.

	**ChR2-evoked IPSC**	**Spontaneous IPSC**	**Electrically-evoked IPSC**
Rise tau (ms)	0.79±0.06	0.89±0.06	0.97±0.13
Decay tau (ms)	8.39±1.25	10.01±0.98	10.79±1.34
Half-width (ms)	9.22±0.60	9.77±0.84	11.31±1.16
Peak amplitude (pA)	159.6±65.0	78.3±11.3	196.1±39.0
Area under the curve	1787±425	885±136	2433±514

*The kinetic values for each IPSC event in a given cell were determined and then all events for that cell were averaged. No significant differences were observed between the three classes of IPSCs for any measure (One-way ANOVA). Data shown are from 8 CeM cells with ChR2-evoked currents and 10 additional cells with electrically evoked IPSCs < 200 pA (peak amplitude approximately matching those of ChR2-evoked currents, since larger IPSCs exhibit longer half-widths), with spontaneous IPSCs determined from IPSC events in the same traces except at the time of evoked IPSCs (at 111 ms into the 1 s sweep)*.

### Chemogenetic activation of CeL CRF neurons induces CRF1 receptor-dependent c-Fos expression in the CeA

**Figure 7:**
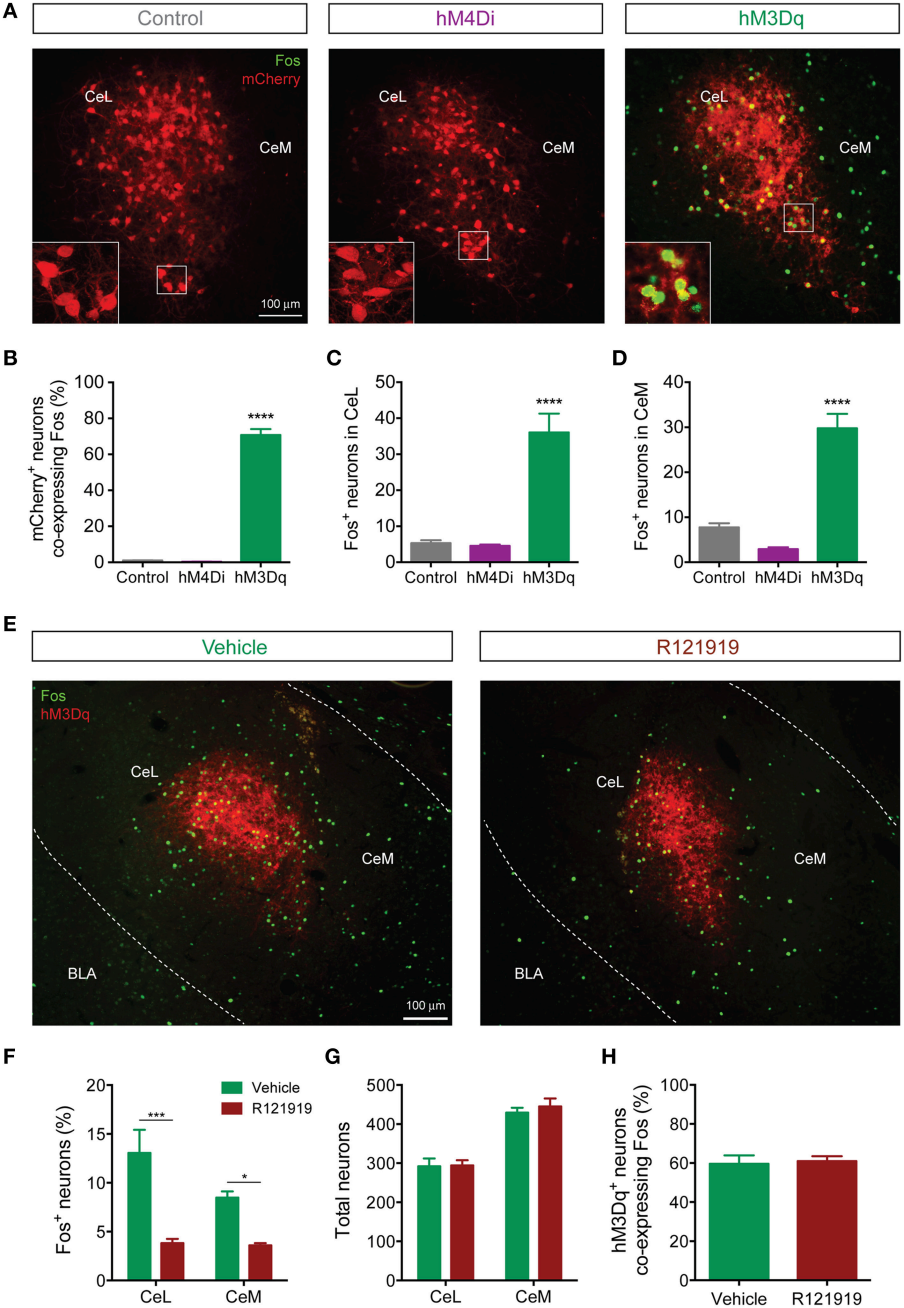
**CeL CRF neurons activate non-CRF neurons in the CeL and CeM**. **(A)** Representative overlay images of Fos immunoreactivity and mCherry fluorescence in control, hM4Di, and hM3Dq-expressing CeL neurons. Scale bar, 100 μm. Insets show high-magnification examples of mCherry expressing neurons immunostained for Fos. **(B)** Percentage of mCherry+ neurons co-expressing Fos following administration of CNO. **(C,D)** Fos induction in non-CRF neurons of the CeL **(C)** and the CeM **(D)** after administration of CNO. ^****^p < 0.001 compared with Control or hM4Di, *n* = 4–5 rats, 10 sections/rat, Tukey's multiple comparisons test. **(E)** Representative overlay images of Fos immunoreactivity and native mCherry fluorescence in hM3Dq-expressing cells from vehicle- or R121919-treated rats. Scale bar, 100 μm. **(F)** Percentage of Fos+ neurons in the CeL and CeM after administration of R121919. **(G)** Total neuron counts per amygdala section are equivalent between groups. **(H)** Percentage of hM3Dq neurons expressing Fos after administration of CNO is equivalent between groups. ^***^p < 0.001, ^*^p < 0.05, *n* = 4–5 rats, 10–12 sections/rat, Tukey's multiple comparisons test.

Since the *Fos* promoter is rapidly induced in strongly activated neurons, *Fos* mRNA and Fos protein are commonly used as surrogate markers of recent neuronal activation (Kaczmarek and Chaudhuri, 1997). To identify patterns of activation downstream of CeL CRF neurons, we examined Fos immunoreactivity following activation of Designer Receptors Exclusively Activated by Designer Drugs (DREADDs) expressed in CeL CRF neurons. Systemic administration of clozapine-N-oxide (CNO, 2 mg/kg) induced Fos expression (Figures 7A,B) in CRF CeL neurons expressing hM3Dq-mCherry, but not in CRF neurons expressing hM4Di-mCherry or mCherry alone [*F*_(2, 10)_ = 351, *p* < 0.0001]. These results suggest that CeL CRF neurons are relatively inactive at baseline. We also found substantial induction of Fos in non-CRF neurons (Figures 7C,D) throughout the CeL [*F*_(2, 10)_ = 27, *p* < 0.0001] and in some cells of the CeM [*F*_(2, 10)_ = 43, *p* < 0.0001]. In the CeL, the proportion that expressed Fos was less than the proportion that exhibited IPSCs after ChR2 stimulation, while in the CeM these proportions were similar (compare Figure 7F and Figure 6E).

Since CeL CRF neurons are GABA-ergic, we were surprised to find that DREADD stimulation excited a subpopulation of CeA neurons. We hypothesized that CRF released onto local CRF1 receptors was responsible, since CRF1 receptors are expressed in the CeA (Van Pett et al., 2000), and in the mouse CeL CRF1 receptor activation enhances spontaneous glutamate release (Silberman and Winder, 2013). To test this hypothesis, we treated rats with 10 mg/kg of the selective CRF1 receptor antagonist R121919 (Chen et al., 2004) prior to activation of hM3Dq. This treatment reduced Fos expression in non-CRF neurons in both CeL and CeM [*F*_*drug*(1, 14)_ = 40.13, *p* < 0.0001] (Figures 7E,F). Importantly, the total number of neurons (Figure 7G) and the proportion of hM3Dq-mCherry positive CRF neurons expressing Fos were similar between R121919 and vehicle-treated groups, indicating that the hM3Dq-driven activity of CeL CRF neurons was not impaired by CRF1 receptor blockade [*p* = 0.786, *t*_(7)_ = 0.282; Figure 7H]. These results demonstrate that stimulation of CeL CRF neurons with hM3Dq excites a subpopulation of non-CRF neurons in the CeL and CeM in a CRF1 receptor-dependent manner, presumably through local release of CRF.

## Discussion

Until recently our knowledge about the anatomy of CRF systems has rested on traditional neuroanatomical methods and inferences about CRF function through administration of drugs that act at CRF receptors. To gain more direct access to CRF neurons to study their functional neuroanatomy we generated a novel BAC transgenic *Crh*-Cre rat. Using Cre-dependent reporters we found Cre recombinase expression in neurons of the CeL and the dorsolateral BNST. There was strong concordance of Cre-dependent transgene expression and CRF immunoreactivity in the CeL, indicating lack of ectopic expression of Cre recombinase in this area. CRF CeL projections were similar to targets of CRF CeL cells identified in previous neuroanatomical studies. However, little is known about their local projections within the CeA, and we found that CRF CeL neurons projected to other non-CRF CeL cells, and also to a smaller number of CeM neurons. These intra-CeA CRF projections exhibited both inhibitory effects, indicated by evoked GABA currents, and excitatory effects, indicated by increased Fos expression, which were prevented by blocking CRF1 receptors. These findings indicate that CRF CeL neurons are a mixed population of interneurons and projection neurons that encode both inhibitory and excitatory information.

Although we found CRF neurons in the CeL and dorsal BNST, CRF cells were absent from the ventral BNST, PVN, and other brainstem and forebrain regions where CRF neurons have been reported (Merchenthaler, 1984; Wang et al., 2011). Thus, despite the size of our BAC vector (~224 kb), Cre expression was limited to two major CRF cell populations, possibly due to incomplete capture of all regulatory elements in the integrated BAC transgene. Although we do not know the precise reason for restricted CRF expression in our animals, it is notable that CRF neurons of the dorsolateral BNST and the CeL share several common features, including medium spiny neuron morphology (Cassell and Gray, 1989; Phelix and Paull, 1990; Sun and Cassell, 1993), expression of the phosphatase STEP (Dabrowska et al., 2013b), and production of GABA (Cassell et al., 1999; Day et al., 1999; Dabrowska et al., 2013b). In contrast, PVN CRF neurons produce glutamate (Dabrowska et al., 2013a) and ventral BNST CRF neurons may also be glutamatergic (Dabrowska et al., 2013a). The *Crh* gene also is regulated differently in these populations of neurons. For example, while corticosterone suppresses CRF expression in the PVN, it up-regulates expression in the CeA and dorsolateral BNST (Swanson and Simmons, 1989; Makino et al., 1994). This differential regulation could involve PKC signaling since we previously found that production of pro-CRF mRNA and protein in the CeA, but not in the PVN, is impaired in PKC epsilon knockout mice (Lesscher et al., 2008). Additionally a recent study identified novel CRF expressing neurons in the VTA, but this expression was only detectable in animals undergoing nicotine withdrawal (Grieder et al., 2014). The detailed mechanisms responsible for heterogeneity in phenotypic characteristics and control of CRF expression among subpopulations of CRF neurons remain to be explored, but the present findings suggest our *Crh*-cre rats may prove useful for selective study of one major subtype of CRF neurons.

**Figure 8:**
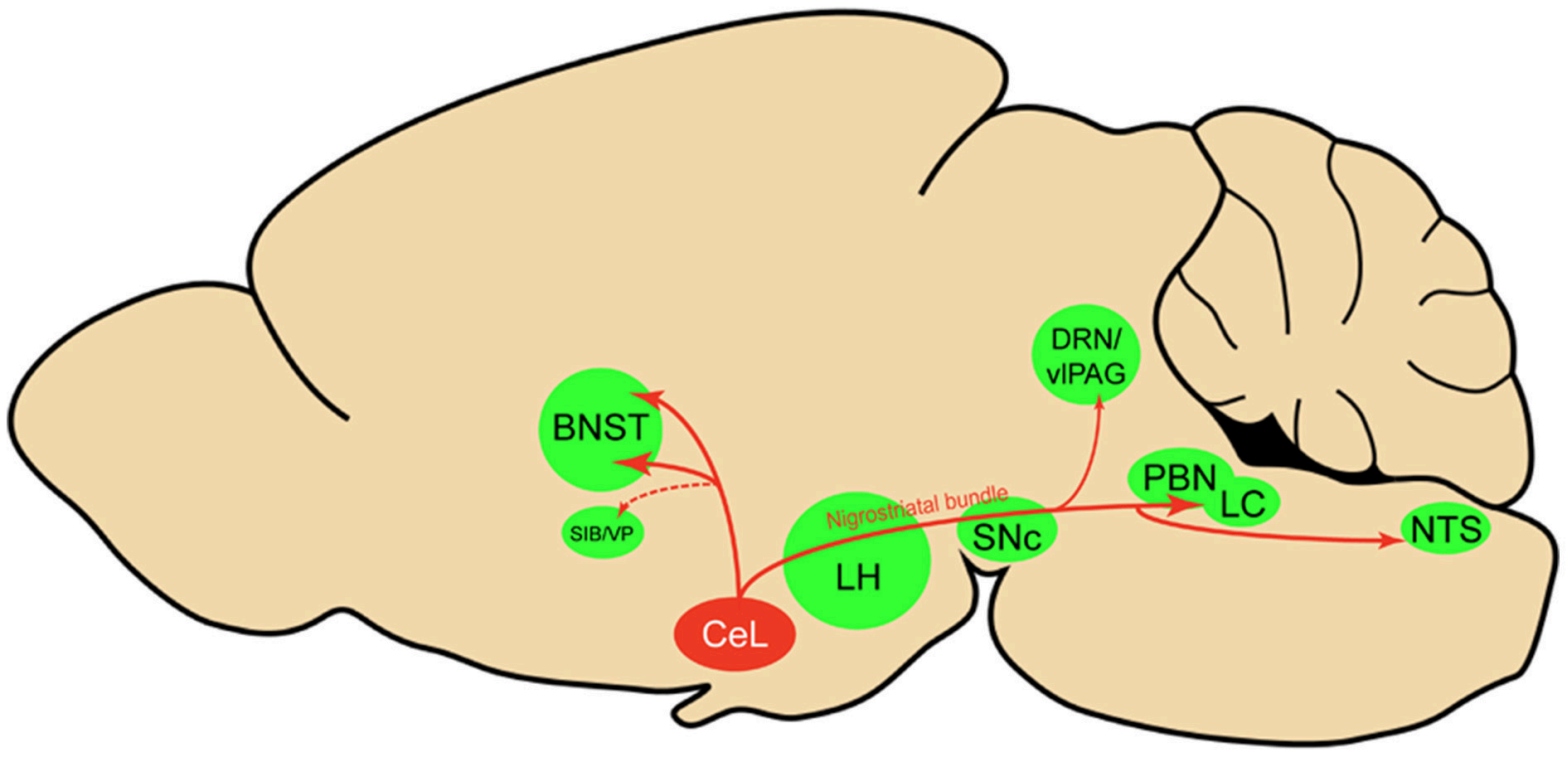
**Sagittal rat brain schematic of CeL CRF neuron projections**. BNST, bed nucleus of the stria terminalis; CeL, lateral central amygdala; DRN, dorsal raphe nuclei; LC, locus coeruleus; LH, lateral hypothalamus; NTS, nucleus tractus solitarius; PBN, parabrachial nucleus; SIB, Substantia innominata; SNc, substantia nigra pars compacta; vlPAG, ventrolateral periaqueductal gray; VP, ventral pallidum.

Using viral delivery of Cre-dependent reporters to identify CeL CRF neurons, we found robust CRF projections from CeL to the brainstem, terminating in the medial and lateral PBN and the LC (Figure 8). There were also extensive projections to the diencephalon, terminating in the dorsal and ventral BNST and the LH. This pattern of connectivity concurs with previously reported CeA CRF projections in the rat (Moga and Gray, 1985; Sakanaka et al., 1986; Van Bockstaele et al., 1998; Reyes et al., 2011). We did not, however, observe projections to the pontine reticular nuclei, as reported in earlier neuroanatomical tracing studies (Gray and Magnuson, 1992; Fendt et al., 1997). A previous study of neuropeptide afferents from the CeA found CRF neurons in the CeL that contained retrograde tracer after injections into the dorsal vagal complex (Gray and Magnuson, 1987). Our results refine this finding by demonstrating that CeL CRF fibers specifically innervate the NTS. Since the NTS provides noradrenergic input to the extended amygdala that plays a role in drug withdrawal and anxiety (Smith and Aston-Jones, 2008), it will be interesting to determine whether a reciprocal connection between CeL CRF neurons and the NTS exists, and whether this circuit is recruited during withdrawal states.

Within the CeA, despite extensive CeL innervation of the CeM (Petrovich and Swanson, 1997), we surprisingly found few CeL CRF projections to the CeM. Instead, we found that CeL CRF neurons preferentially innervate other CeL neurons. Given the sparseness of projections to CeM, we speculate that CeL CRF projection neurons act in parallel with CeM projection neurons to regulate behavior, and that the small number of direct CeL CRF projections to the CeM, and potentially more extensive indirect projections *via* non-CRF neurons in the CeL, coordinate the actions of CeL and CeM systems on behavior. Interesting questions for the future are whether individual CeL CRF neurons project to many or a restricted set of targets and whether the same or different CeL CRF neurons serve as interneurons and projection neurons. Future studies using *Crh*-Cre rats and Cre-dependent tracing tools and actuators should allow us to unravel this circuitry in greater detail.

Despite CeL CRF neurons being GABA-ergic, activation of the excitatory DREADD hM3Dq in these neurons induced expression of Fos in several non-CRF neurons of the CeL and CeM. Thus, CRF CeL neurons can generate both excitatory (Fos) and inhibitory (GABA IPSCs) responses in CeL and CeM neurons. Fos induction following activation of CRF CeL neurons involved CRF release since it was substantially reduced by administration of a CRF1 receptor antagonist. Depending on the synapse, activation of CRF1 receptors can activate neurons by enhancing glutamatergic transmission. For example, in the rat lateral capsular CeA, CRF acting at CRF1 receptors enhances glutamatergic transmission from parabrachial efferents (Ji et al., 2013), and CRF increases the frequency of spontaneous EPSCs in the mouse CeL through actions at CRF1 and CRF2 receptors (Silberman and Winder, 2013). The actions of CRF on excitatory neurotransmission in the rat CeL have yet to be determined.

Given our limited knowledge of intra-CeA circuitry, we can envision several mechanisms by which activating CRF CeL neurons could generate both inhibitory and excitatory responses. First, GABA and CRF may affect different target neurons, with GABA released at synapses and CRF released non-synaptically to signal via local volume transmission that results in excitation of CeA neurons through convergent disinhibition and CRF signaling. A somewhat similar situation has been recently described for innervation of the cerebral cortex and striatum by histaminergic neurons of the hypothalamus that also release GABA (Yu et al., 2015). Alternatively, excitatory effects of CRF may be partly suppressed by concurrent GABA release, for example where CRF acting at CRF1 receptors enhances GABA release, as has been demonstrated in the rat CeM (Herman et al., 2013). On the other hand, in cells having a depolarized Cl^−^ reversal potential, activation of GABA_*A*_ receptors could synergize with CRF to directly activate post-synaptic neurons (Staley and Proctor, 1999), although this may be more speculative for adult neurons. Finally, stimulus intensity and duration may affect GABA and CRF release differently, leading to a range of inhibitory and excitatory responses on the same target neuron population. Future optogenetic and chemogenetic studies using *Crh*-Cre rats could help to determine if the actions of GABA and CRF occur at the same or at different neurons and to elucidate mechanisms by which these transmitters act.

The generation of several *Crh*-Cre mouse lines has facilitated our understanding of CRF circuits and their roles in several behavioral states. At least three *Crh*-Cre mouse lines have been reported, and have been used to demonstrate roles for CRF neurons in fear conditioning (Gafford et al., 2014), fear extinction (Gafford et al., 2012), anxiety and avoidance behaviors (Gafford et al., 2012; McCall et al., 2015), and binge-like alcohol consumption (Pleil et al., 2015). However, a recent review (Chen et al., 2015) indicates that two of these lines, the *Crh*-BAC transgenic (Alon et al., 2009) and CRFp3.0Cre (Martin et al., 2010) exhibit ectopic Cre transgene expression, whereas *Crh*-IRES-Cre mice (Taniguchi et al., 2011) express Cre with high fidelity to endogenous CRF across the brain. Since our current rat line is a BAC transgenic, our determination of Cre and CRF fidelity in the amygdala (Figure 1) was critically important. Although our rat shows limited expression of Cre, it provides a tool to study the role of GABAergic CRF neurons of the amygdala and dorsolateral BNST to not only complement work done with *Crh*-Cre mice, but to permit investigation of more complex behaviors such as operant conditioning and sophisticated learning tasks that cannot easily be studied using mice.

The anatomy of projections from CeL to CeM has been examined recently in mouse models of fear conditioning (Ciocchi et al., 2010; Haubensak et al., 2010; Li et al., 2013), although without a focus on amygdala CRF neurons or employing *Crh*-Cre mice. An anatomical framework has emerged in which fear-related cues excite BLA neurons, which in turn enhance firing in a CeL cell subpopulation termed On-cells that inhibit a separate CeL subpopulation termed Off-cells, the net result of which is to disinhibit CeM neurons. The subsequent increase in CeM activity mediates conditioned fear *via* projections downstream to somatic and autonomic brainstem nuclei. In mice the PKCδ neuron subpopulation in the CeL represents Off-cells (Haubensak et al., 2010), which tonically suppress CeM neurons to inhibit fear responses. On-cells in mice are at least partially SOM+ (Li et al., 2013). In contrast, the role of CeL CRF neurons in this fear control circuit has been largely overlooked. Here we provide evidence consistent with rat CRF neurons being mostly a subpopulation of On-cells based on co-expression of SOM in about 40% of CRF neurons and sparse projections to the CeM. Future challenges will be to dissect the relative contribution of CRF neurons (SOM+ and SOM-) to fear-related circuitry and behavior, and to determine whether CRF neurons with unique neuropeptide co-expression profiles provide distinct inputs to local and distant projection targets.

In summary, we present a novel transgenic Cre driver rat line that permits selective targeting of CRF-expressing GABAergic neurons of the extended amygdala. The *Crh*-Cre rat will be an important tool for dissecting extended amygdala CRF systems in the control of fear and anxiety, as well as stress-sensitive behaviors, such as feeding and drug seeking. Furthermore, species-specific phenotypic differences can be evaluated by comparing *Crh*-Cre rats with *Crh*-Cre mice, which should help unify conclusions about CRF circuits across rodent species.

## Author contributions

Study concept and design: MP, EM, FH, PJ, RM. Acquisition of data: MP, EM, FH, RK, RM, AB, VK, GD, EC. Analysis and interpretation of data: MP, EM, FH, GD, OG, RM. Drafting of the manuscript: EM, FH. Critical revision of the manuscript for important intellectual content: MP, RM. Statistical analysis: MP, FH. Obtained funding: FH, OG, PJ, RM. Administrative, technical, and material support: EC, KR. Study supervision: FH, PJ, RM.

## Funding

This work was supported by pilot project funds through U01 AA013517 to FH., grants AA13588 and AA017072 to RM, and AA020608, AA006420 and AA022977 to OG, and funds provided by the State of California for medical research for alcohol and substance abuse through UCSF to PJ and RM. A portion of this work supported by the National Institute on Alcohol Abuse and Alcoholism and the National Institute on Drug Abuse Intramural Research Programs. MP is supported by Graduate Research Fellowship DGE-1110007 from the National Science Foundation.

### Conflict of interest statement

The authors declare that the research was conducted in the absence of any commercial or financial relationships that could be construed as a potential conflict of interest.
